# Calculation of incubation period and serial interval from multiple outbreaks of Marburg virus disease

**DOI:** 10.1186/1756-0500-7-906

**Published:** 2014-12-13

**Authors:** Boris I Pavlin

**Affiliations:** General Preventive Medicine Residency Program, Johns Hopkins Bloomberg School of Public Health, 615 N Wolfe St, Baltimore, MD 21205 USA; Office of the WHO Representative in Papua New Guinea, World Health Organization, PO BOX 5896, Boroko, NCD 111 Papua New Guinea

**Keywords:** Marburg, Clinical onset serial interval, Incubation period, Outbreak, Haemorrhagic fever

## Abstract

**Background:**

Marburg viruses have been responsible for a number of outbreaks throughout sub-Saharan Africa, as well as a number of laboratory infections. Despite many years of experience with the viruses, little is known about several important epidemiologic parameters relating to the development of Marburg virus disease. The analysis uses pooled data from all Marburg cases between 1967 and 2008 to develop estimates for the incubation period and the clinical onset serial interval (COSI).

**Methods:**

Data were obtained from original outbreak investigation forms (n = 406) and from published data (n = 45). Incubation periods were calculated for person-to-person exposure, for laboratory-acquired infections, and for presumed zoonotic exposures. Similar analysis was conducted for COSI, using only cases with unambiguous person-to-person transmission where both the primary and the secondary case patients had well-defined illness onsets.

**Results:**

Seventy-six cases were retained for the incubation period analysis. Incubation periods ranged from a minimum of 2 days in the case of two laboratory workers to a maximum of at least 26 days for a person-to-person household transmission. Thirty-eight cases were retained for COSI analysis. The median COSI was 11 days, with an interquartile range of 8 to 15.

**Conclusions:**

This study extends the maximum known incubation period of Marburg virus disease to 26 days. The analysis was severely hampered by a lack of completeness in epidemiologic data. It is necessary to prioritize obtaining more accurate epidemiologic data in future outbreaks; greater use of COSI may facilitate an improved understanding of outbreak dynamics in Marburg and other diseases.

## Background

Marburg virus disease was the first identified filovirus haemorrhagic fever, caused by two clinically indistinguishable enveloped single-stranded RNA viruses (Marburg virus and Ravn virus) [1] closely related to the Ebola viruses. It was first discovered in 1967 during an outbreak of haemorrhagic fever among laboratory staff in Marburg and Frankfurt, Germany and Belgrade, Serbia (then Yugoslavia) who had contact with Ugandan vervet monkeys (*Cercopithecus aethiops*) [2]. Since that time, Marburg viruses have been responsible for a number of outbreaks throughout sub-Saharan Africa, as well as a number of laboratory infections (Table 1). Symptoms begin with a non-specific influenza-like-illness (e.g., fever, headache, myalgias) which may proceed to haemorrhagic manifestations, with death occurring in roughly 20–90% of cases [3].

**Table 1:** **History of Marburg outbreaks, 1967–2008**

Outbreak	Circumstances	Total cases	Deaths (CFR)	Source of data
**Germany and Yugoslavia ex. Uganda, 1967**	Infected laboratory primates imported from Uganda; lab workers and contacts developed illness	31	7 (23%)	[2, 4]
**South Africa ex. Zimbabwe, 1975**	Australian traveler to Zimbabwe visited South Africa and developed symptoms; infected traveling companion and South African nurse	3	1 (33%)	[5]
**Kenya, 1980**	Visitor to Kitum cave died; infected doctor, who survived	2	1 (50%)	[6]
**Kenya, 1987**	Visitor to Kitum cave died	1	1 (100%)	[7]
**Russia, 1988**	A laboratory worker died after a needlestick at ‘Vektor’ bioweapons research facility	1	1 (100%)	[8]
**Russia, 1990**	A laboratory worker was infected after a needlestick at Vektor, but survived	1	0 (0%)	[9]
**DR Congo, 1998–2000**	Outbreak originating among miners in a gold mine; spread to community; sporadic cases for years after main outbreak ceased	154	128 (83%)	Primary data
**Angola, 2004–2005**	Urban outbreak amplified through nosocomial transmission	252	227 (90%)	Primary data
**Uganda, 2007**	Informal gold miners in Kamwenge District, Uganda infected; infection in mine +/− person-to-person transmission	4	2 (50%)	[3]
**Colorado ex. Uganda, 2008**	U.S. traveler visited ‘Python’ cave in Maramagambo forest in Uganda; developed illness upon return to U.S.	1	0 (0%)	[10]
**Netherlands ex. Uganda, 2008**	Dutch traveler visited ‘Python’ cave in Maramagambo forest in Uganda; developed illness upon return to Netherlands	1	1 (100%)	[11]
**GRAND TOTAL**		451	369 (82%)	

*CFR* Case Fatality Ratio.

Despite many years of experience with the viruses, little is known about several important epidemiologic parameters relating to the development of Marburg virus disease. The incubation period, one of the most basic parameters, has been estimated as being up to 21 days (typically 5–10 days) primarily from the laboratory-acquired infections that occurred in 1967 in Europe; what little information has been published about Marburg infections in the natural setting in Africa has been gleaned from isolated cases or small outbreaks (Table 1). This analysis used pooled data from all Marburg cases between 1967 and 2008 (the last known occurrence), including unpublished data from the 1998–1999 Democratic Republic of the Congo outbreak and the largest-ever 2004–2005 Angola outbreak, to develop estimates for the clinical incubation period and the clinical onset serial interval (COSI; a validated epidemiologic measurement defined as the time between the onset of symptoms of the primary case and the onset of symptoms of the secondary case)^a^ [12]. These data add to the epidemiologic knowledge of Marburg disease and may help inform future outbreak investigations.

## Methods

### Sources of data

Data were obtained from published case reports (n = 45) for outbreaks other than the 1998–1999 Democratic Republic of the Congo and the 2004–2005 Angola outbreaks (Table 1). Data from the latter outbreaks (n = 406) were obtained from case investigation forms obtained by the author. Only cases classified as Probable^b^ or Confirmed were retained. Thus, of the 451 known cases, 180 were retained (43 Probable and 137 Confirmed). The sub-analyses described below were performed on smaller samples because not all data were available for all patients.

### Incubation period analysis

The criteria for determining the incubation period in person-to-person exposure were as follows: patient pairs were chosen in which a secondary case could be linked to another patient in the database. At least one of these patients must have been lab-confirmed. Also, neither case could have been specifically ruled out for Marburg, usually by a negative lab result and plausible alternative diagnosis. When an exact exposure event was not known, an incubation period range was calculated based on the first and last contact with the primary case.

For laboratory-acquired infection, incubation period was calculated as the time from a known exposure incident to Marburg virus until onset of symptoms. Where exact exposure could not be determined, incubation period was calculated from last known work with infectious virus until onset of symptoms.

For presumed zoonotic infection (e.g., isolated cave/mine incursions or contact with dead animals), incubation period was calculated from time of exposure to the zoonotic source, if its date could be identified precisely, until onset of symptoms. In one case (Netherlands ex. Uganda, 2008), the exact exposure was not clear but was strongly suspected to be a specific cave incursion [11].

Data were entered into STATA/SE 11.0 (Stata Corp., College Station, TX).

### Clinical outcome serial interval (COSI)

Because of the inherent uncertainty in determination of incubation period, the analysis above was repeated using the COSI. Only cases with unambiguous person-to-person transmission were retained; hence, certain cases such as those involving three miners in Uganda in 2007 [3] were discarded. Both the primary and the secondary case were required to have well-defined illness onsets.

## Results

Many cases, especially from the 1967 outbreak, were not described in sufficient detail to allow thorough analysis. Additionally, most case investigation forms from DR Congo and Angola were only partially completed. Data from Probable and Confirmed patients were pooled for further analysis.

Among the 154 cases for whom age was available, the median age was 30 years (range <1 to 88 years). Primary (n = 41) cases were significantly older (median 35 years) than secondary (n = 31) cases (median 28 years) (Wilcoxon rank-sum test, p = 0.0011).

58% of the cases were female. There were significantly fewer females among primary cases (37%) than among the secondary cases (77%) (Z-test, p = 0.0006).

The overall case fatality ratio (n = 149) was 66%; there was no significant difference between primary and secondary cases.

### Incubation period

76 cases were examined for this analysis. 53% were female. The median age (n = 75) was 30, with an interquartile range of 20 to 43, statistically equivalent to the analyzed cases as a whole. The case fatality ratio was 47%.

Incubation periods ranged from a minimum of 2 days (in the case of two laboratory workers from Marburg for whom precise exposures were known) to a maximum of at least 26 days (in a woman from Uige who had extended contact with an unconfirmed case, the last contact being 26 days prior to her symptoms), or 23 days (in the case of the infant child of a confirmed case, with whom the last contact was 23 days prior to his illness).

Only 18 cases had precise exposure dates that allowed for calculation of incubation period statistics. Of these, 4 were categorized as exposures to laboratory animals, 4 were healthcare exposures, 4 were cave or mine incursions, 3 were exposures to cell culture, 2 were patients from Uige with single exposures such as attending a funeral, and one was sexual exposure to a recovered partner. Overall, the median incubation period was 7 days, with a range of 2 to 13 days; there was no significant difference between primary and secondary cases (Wilcoxon rank-sum test, p = 0.47). The small number of cases in each category precluded meaningful analysis by exposure category.

### Clinical Onset Serial Interval (COSI)

The expectation was that using the COSI would provide for a greater sample size for analysis than did the incubation period; this turned out not to be the case. The number of samples included in this analysis was thus 38 (22 from Angola, 12 from the Democratic Republic of the Congo, 2 from South Africa, and 1 each from Serbia and Kenya), reflecting 76 primary-secondary pairs. The median COSI was 11 days, with an interquartile range of 8 to 15.

## Discussion

This study attempted to combine data from a variety of Marburg virus outbreaks for the purposes of evaluating incubation period and clinical onset serial interval (COSI).

In the first analysis, the median incubation period was 7 days. Interestingly, there was no significant difference in incubation period observed in secondary versus primary cases, despite the initial suggestion from the 1967 cases that this was the case. This may be because, in the initial laboratory infections, there was an overwhelmingly large difference in inoculum between the primary laboratory exposures and the secondary person-to-person exposures; in later primary exposures, exposure was more often to an animal or mine, where the inoculum would be expected to be significantly lower. The low sample size precludes testing this hypothesis.

Also, unlike the original case reports, there was no difference in case fatality ratio between primary and secondary cases (possibly because of the same inoculum effect described above). Primary cases were older and more likely to be male than secondary cases, likely related to the exposure setting (i.e., the definition of ‘primary’ indicates an exposure such as laboratory work, hunting or mining, activities likely to be undertaken by adult males, whereas ‘secondary’ exposure is largely in the domestic setting, where contacts are more likely to be young and female).

An important finding in the incubation period analysis was that two patients had incubation periods longer than the accepted^c^ 21-day maximum incubation period of Marburg disease. The patient with the longest incubation period, a *minimum* of 26 days, was a 52-year-old female with prolonged contact with an uninvestigated case patient. It is possible that the precise date of the primary patient’s death, being based on the secondary case’s recall, was misremembered. On the other hand, the patient with a 23-day incubation period was an infant male with laboratory-confirmed Marburg infection whose father was a nurse who died of suspected Marburg infection (as he died early in the course of the outbreak, no laboratory testing was performed) and was investigated. The infant’s incubation period was calculated from the known date that his father died (i.e., last exposure), so 23 days again represents a minimum estimate. While later acquisition of Marburg virus from a source other than his father is theoretically possible, it is less likely, as he did not have contact with any other known or suspected cases. These findings may have operational implications, as 21 days is used in outbreak responses as the maximum period when defining exposure risk factors (e.g., ‘attendance at a funeral in the past 21 days’), and it is also used as the basis for declaring the end of a Marburg outbreak (42 days, i.e., two incubation periods, since the last known case). On the other hand, it has already been observed that the maximum incubation period for Ebola, while usually claimed to be 21 days, may be up to 25 days [13]; yet there do not seem to have been any major public health implications to date as a result of retaining the shorter operational period. In any case, it is expected that most cases will fall within the more familiar 21-day window [14]. Further attention should be paid to the maximum incubation period in future Marburg outbreaks, and changes to the operational maximum incubation period made if warranted by empirical observation of any transmission beyond 21 days, weighed against the significant operational strain of expanding the operational incubation period to pick up the small number of additional cases.

In the COSI analysis, the median COSI was 11 days. The COSI is notable for its robustness in the face of imprecisely-defined exposures, such as may occur when a family member has repeated exposures while caring for an infected person; while many people will be unable to determine when they may have been infected, few people forget when a severe acute illness began. The implication of there being a median COSI of 11 days versus a median incubation period of 7 days is that an infection is most likely to be transmitted around the 4th day of illness; the reason for this are likely a complex interplay between virus kinetics and patient behaviour.

The greatest limitation to these analyses is the low sample sizes involved. In earlier cases, original records were unavailable; however, even in cases where original records were available (i.e., the D.R. Congo and Angola outbreaks), most forms were woefully incomplete, leading to multiple missing data for a given variable (for example, less than 10% of Angola cases were complete enough to calculate the incubation period). This was compounded by the existence of different forms, which often contained different variables, from different periods of an outbreak.

An additional challenge in interpreting data lies with the ambiguity of certain questions. For example, the question “were you hospitalized” could potentially be a useful proxy for illness severity (if meant to indicate hospitalization after disease onset), or could indicate risk of nosocomial infection (if meant to indicate hospitalization prior to disease onset); since the intent of the question was to assess for nosocomial infection, this question would have been clearer if it had been phrased “in the period of 1 week to 1 month prior to your illness, were you hospitalized?”. Other examples of un-interpretable questions included open-ended questions, such as “what type of contact did you have with a suspected case?” (This produced a wide variety of answers, including “direct,” persons’ names, relationships, and activities). Open-ended questions have their place early in the outbreak, for exploratory purposes; however, they must quickly be supplanted by questions whose answers are quantifiable.

During the design of questionnaires, it is also important to ask “what am I going to do with the results of this question?” (In other words, is the question actionable?). If no clear action is suggested by the results of a question, it should be omitted. The exception is the inclusion of questions designed to answer research questions (for example, exposure to wild animals as possible reservoirs). In the long run, taking the time to answer these research questions in the field may actually save resources by preventing future epidemics.

Setting aside a few hours at the beginning of an outbreak to thoughtfully design a questionnaire containing quantifiable and actionable questions is well worth the time. In the heat of the moment, methodical descriptive epidemiology often takes second priority to such activities as infection control and case finding; unfortunately, without data (and their real-time analysis), such activities are often misguided and unsuccessful. It is equally important to make sure that once a question is included on a questionnaire, it is asked and the results are recorded. While the questions needed in each outbreak are different, many of the principles, and even some of the questions, are universal. It would be useful for the World Health Organization to develop a “minimum standard” for the collection of data during an outbreak, to be ignored at one’s peril. These issues are not dissimilar to the problem of poor clinical documentation during outbreaks of Marburg and other haemorrhagic fevers [15, 16].

Finally, it is important to expand the use of the COSI as an important epidemiologic tool for outbreak assessment and action. It can be a useful tool for identifying the pathogen, and evaluating the success of control interventions. The main advantage of the COSI is that it is more easily measurable than the incubation period (as patients are more likely to know when they became ill than when they were exposed). Unfortunately, as an epidemiologic tool, it currently has limited use because a disease’s published incubation period is often used to help identify the pathogen, but few diseases have published COSIs (one notable exception is influenza [17, 18]), and calculation of the COSI from available statistics is difficult. The scientific and public health community should endeavor to characterize the COSIs of important outbreak-prone diseases, as well as look into ways to operationalize the use of COSI in outbreak response.

## Conclusions

This study extends the maximum incubation period of Marburg virus disease to 26 days. The analysis was severely hampered by a lack of completeness in epidemiologic data. In order to better characterize the epidemiology of Marburg, it will be necessary to prioritize obtaining more accurate exposure and clinical history data in future outbreaks; greater use of COSI may facilitate an improved understanding of outbreak dynamics in Marburg and other diseases.

## Endnotes

^a^The COSI depends on three things: the interval between the time of primary case’s infection and the beginning of infectiousness (not necessarily the beginning of symptoms); the duration of infectiousness; and the time from infection of the secondary case until clinical disease onset, also known as the incubation period. The COSI for a given pathogen can change depending on circumstances, much as with incubation period. For instance, it is altered by more severe disease, which for example can lead to earlier infectiousness and therefore a shorter COSI. It is also altered by successful control measures, because the shorter duration of infectiousness (due to recovery or isolation) leads to a shorter COSI.

^b^The definition of ‘probable’ varied slightly from one outbreak to another, but primarily involved a clinically compatible syndrome with an epidemiologic link to a laboratory-confirmed case.

^c^There appears to be no objective evidence for this maximum incubation period for Marburg disease, except extrapolation from the maximum incubation period for Ebola, which was reported as 21 days by Breman in 1977 [19].

## Authors’ information

Dr. Pavlin is a preventive medicine physician who has spent his career working in emerging disease epidemiology and outbreak control. This work was conducted as part of his preventive medicine residency training. He is currently an emerging disease surveillance and response epidemiologist with the World Health Organization in Papua New Guinea. The views expressed in this article do not represent the views of the World Health Organization.
